# Diagnostic effectiveness of quantitative [^18^F]flutemetamol PET imaging for detection of fibrillar amyloid β using cortical biopsy histopathology as the standard of truth in subjects with idiopathic normal pressure hydrocephalus

**DOI:** 10.1186/2051-5960-2-46

**Published:** 2014-04-22

**Authors:** Ville Leinonen, Juha O Rinne, Dean F Wong, David A Wolk, John Q Trojanowski, Paul F Sherwin, Adrian Smith, Kerstin Heurling, Mandy Su, Igor D Grachev

**Affiliations:** 1 Department of Neurosurgery, Kuopio University Hospital NeuroCenter and Institute of Clinical Medicine, University of Eastern Finland, Kuopio, Finland; 2 Turku PET Centre and Department of Neurology, University of Turku and Turku University Hospital, Turku, Finland; 3 The Russell H. Morgan Department of Radiology and Radiological Science, Psychiatry and Neuroscience, Johns Hopkins University School of Medicine, Baltimore, MD, USA; 4 Department of Neurology, Penn Memory Center, University of Pennsylvania, Philadelphia, PA, USA; 5 Department of Pathology, Penn Memory Center, Institute on Aging, and Center for Neurodegenerative Disease Research, University of Pennsylvania, Philadelphia, PA, USA; 6 Life Sciences, GE Healthcare, Princeton, NJ, USA; 7 Life Sciences, GE Healthcare, Amersham, UK; 8 Life Sciences, GE Healthcare, Uppsala, Sweden; 9 Biostatistics, H2O Clinical, Hunt Valley, MD, USA; 10 Life Sciences, GE Healthcare, 101 Carnegie Center, Princeton, NJ 08540, USA

**Keywords:** Alzheimer’s disease, PET, Brain biopsy, [^18^F]flutemetamol, Fibrillar amyloid β, Normal pressure hydrocephalus

## Abstract

**Introduction:**

PET imaging of amyloid-β (Aβ) in vivo holds promise for aiding in earlier diagnosis and intervention in Alzheimer’s disease (AD) and mild cognitive impairment. AD-like Aβ pathology is a common comorbidity in patients with idiopathic normal pressure hydrocephalus (iNPH). Fifty patients with iNPH needing ventriculo-peritoneal shunting or intracranial pressure monitoring underwent [^18^F]flutemetamol PET before (N = 28) or after (N = 22) surgery. Cortical uptake of [^18^F]flutemetamol was assessed visually by blinded reviewers, and also quantitatively via standard uptake value ratio (SUVR) in specific neocortical regions in relation to either cerebellum or pons reference region: the cerebral cortex of (prospective studies) or surrounding (retrospective studies) the biopsy site, the contralateral homolog, and a calculated composite brain measure. Aβ pathology in the biopsy specimen (standard of truth [SoT]) was measured using Bielschowsky silver and thioflavin S plaque scores, percentage area of grey matter positive for monoclonal antibody to Aβ (4G8), and overall pathology impression. We set out to find (1) which pair(s) of PET SUVR and pathology SoT endpoints matched best, (2) whether quantitative measures of [^18^F]flutemetamol PET were better for predicting the pathology outcome than blinded image examination (BIE), and (3) whether there was a better match between PET image findings in retrospective vs. prospective studies.

**Results:**

Of the 24 possible endpoint/SoT combinations, the one with composite-cerebellum SUVR and SoT based on overall pathology had the highest Youden index (1.000), receiver operating characteristic area under the curve (1.000), sensitivity (1.000), specificity (1.000), and sum of sensitivity and specificity for the pooled data as well as for the retrospective and prospective studies separately (2.00, for all 3). The BIE sum of sensitivity and specificity, comparable to that for quantitation, was highest using Bielschowsky silver as SoT for all SUVRs (ipsilateral, contralateral, and composite, for both reference regions). The composite SUVR had a 100% positive predictive value (both reference regions) for the overall pathology diagnosis. All SUVRs had a 100% negative predictive value for the Bielschowsky silver result.

**Conclusion:**

Bielschowsky silver stain and overall pathology judgment showed the strongest associations with imaging results.

## Introduction

Despite the early description of Alzheimer’s disease (AD) in 1906 [1], the first set of globally accepted criteria for the clinical diagnosis of AD was only established in 1984 by the National Institute of Neurological and Communicative Disorders and Stroke and the Alzheimer’s Disease and Related Disorders Association (NINCDS-ADRDA) [2]. Prior to these criteria, a suspected clinical diagnosis of AD could only be confirmed with certainty by the post mortem observation of ‘senile plaques’ and neurofibrillary tangles in the brain, much as they were described to appear by Alois Alzheimer himself as he visualized them with low power microscopy using Bielschowsky silver stain [1]. While diagnosis in living patients represents a significant advance, when applied by expert clinicians the 1984 clinical criteria are reported to have only approximately 80% positive predictive value (PPV) and 60% negative predictive value (NPV) for a pathology-confirmed diagnosis [3]. In general, by clinical criteria, sensitivity (proportion of true positive results) increases with more permissive clinical criteria, and specificity (proportion of true negative results) increases with more restrictive clinical criteria. However, the opposite is true for neuropathologic criteria.

Appropriately, a proposal to develop and validate new biomarkers for AD was included in the 2011 National Institute on Aging-Alzheimer’s Association (NIA-AA) updated diagnostic recommendations [4-7]. Underpinning the 2011 recommendations is the recognition that the progression of AD biomarkers over time likely follows an ordered temporal sequence, and the Aβ biomarkers (e.g., low brain Aβ clearance, i.e., low cerebrospinal fluid [CSF] Aβ_42_; and tracer retention on amyloid positron emission tomography [PET] imaging) are representative of upstream events [5].

While normally CSF flows out of the brain ventricles, idiopathic normal pressure hydrocephalus (iNPH) may be caused by CSF ‘backflow’ into the brain from the ventricles [8]. Studies of various analytes have shown that a concentration gradient exists between CSF taken from the ventricles (higher concentration) and CSF taken from the lumbar spine (lower concentration) [9-12]. Aβ oligomers self-assemble into the larger Aβ species [13], which are deposited as plaque in the brain; elevated soluble Aβ oligomers in CSF have been associated with AD, although the data are inconsistent and the culprit toxic oligomer still needs to be identified [14]. With a hypothetical leap, it may not be so surprising that AD-like Aβ pathology frequently occurs concomitantly with iNPH.

Clinically, patients with iNPH present with a classic triad of symptoms: cognitive impairment, gait abnormalities, and urinary incontinence. Untreated, iNPH is progressive [15]. The treatment for iNPH is surgical placement of a ventriculoperitoneal shunt. A small cortical biopsy from the site of shunt placement may be obtained during the procedure. Though iNPH itself may cause dementia, an identifiable neurodegenerative process often underlies the cognitive decline [16]. Concomitant AD pathology is not uncommon [17-19], up to 68% in one series [20], and may be associated with a poorer response to ventriculoperitoneal shunting [17,18]. Biopsies from iNPH patients represent a unique opportunity to study correlations between PET amyloid imaging and pathology in living subjects.

The PET radioligand, Pittsburgh compound B (PiB), which is a neutral analog of thioflavin T (a stain which detects fibrillar amyloid) has been extensively studied both in living subjects and in autopsy tissue. PiB was designed to cross the blood-brain barrier, bind with high selectivity and nanomolar affinity to fibrillar Aβ, and clear rapidly from the brain [21]. Cortical [^11^C]PiB uptake, as measured by PET in living subjects, correlates with fibrillar Aβ load measured subsequently by immunohistochemical stains for Aβ_40_ and Aβ_42_ post mortem [22-24]. Unfortunately, the short half-life of [^11^C] (about 20 minutes) requires an on-site cyclotron for its production, limiting its use to academic medical and specialized imaging centers. Consequently, several fluorinated amyloid imaging agents have emerged to bridge this gap (F18 having a half-life of about 110 minutes): [^18^F]flutemetamol [25] and [^18^F]florbetapir [26] (Aβ diagnostics both approved by the Food and Drug Administration]) as well as [^18^F]florbetaben making the radiopharmaceutical available for widespread community use.

[^11^C]PiB, [^18^F]florbetapir, and [^18^F]florbetaben have similar fibrillar Aβ binding site affinities, and can be used in a comparable manner to assess brain amyloid density [27]. In this analysis, we used [^18^F]flutemetamol, which differs from PiB in structure by the addition of a single F18 atom [28].

Four clinical studies (GE067-008, -009, -010, and -011, called Studies A, B, C, and D, here (and published separately as [20,29-31], respectively)) were undertaken in iNPH patients requiring surgical shunt procedures or intracranial pressure (ICP) monitoring, to determine how well cerebral fibrillar Aβ uptake of [^18^F]flutemetamol as quantified by PET imaging corresponded with immunohistochemical (IHC) and histochemical (HC) estimates of amyloid burden in biopsy samples taken during these procedures. Two of the studies (one in Europe and one in the United States [US]) were retrospective studies in which the biopsy was followed by the PET scan. Two of the studies (one in the Europe and one in the US) were prospective studies, in which the order of procedures was reversed.

[^18^F]Flutemetamol uptake was measured quantitatively in specific brain regions including the cortical area of (prospective studies) or surrounding (retrospective studies) the biopsy site (*ipsilateral* site) and the site in the contralateral hemisphere that corresponded to the biopsy site (*contralateral* site). A composite neocortical measure of [^18^F]flutemetamol uptake was also calculated by averaging the uptake from frontal cortex, anterior cingulate gyrus, posterior cingulate gyrus/precuneus, lateral-temporal cortex, and parietal cortex. Aβ plaque frequency was determined and scored in biopsy specimens stained with Bielschowsky silver stain and thioflavin S. The percentage of grey-matter area occupied by plaque was also assessed following IHC for the monoclonal antibody 4G8. Finally, based on all available stains/slides, an overall pathology assessment was rendered of the Aβ load in biopsy tissue grey matter. These 4 pathology endpoints served as the standard of truth (SoT) in comparisons with the [^18^F]flutemetamol PET data.

In a pooled analysis of these 4 studies as previously reported, for specific parameters, [^18^F]flutemetamol uptake in ipsilateral and contralateral sites as well as the composite cerebral cortical measure of [^18^F]flutemetamol uptake were significantly correlated with Aβ plaque burden [32]. This confirmed similar findings in an autopsy study using [^18^F]florbetapir [33].

The standard uptake value ratio (SUVR) is a quantitative measure of tracer uptake in a brain, normalized for the mean uptake in a reference region. Pooling data from Studies A, B, C, and D, using biopsy pathology as the SoT and cerebellar grey matter as the quantitative PET reference region in one set of data and pons in another, here we set out to find (1) which pair(s) of PET SUVR and pathology SoT endpoints matched best, (2) whether quantitative measures of [^18^F]flutemetamol PET were better for predicting the pathology outcome than majority [^18^F]flutemetamol PET visual-based image read, and (3) whether there was a better match between PET image findings in retrospective vs. prospective studies.

## Materials and methods

### Patients

Patients were eligible for inclusion if they had known or suspected iNPH, were older than 50 years of age, and were in general health appropriate for study procedures. Patients were excluded if they were pregnant or lactating, had known or suspected hypersensitivity/allergy to the [^18^F]flutemetamol formulation, or had a contraindication to PET or magnetic resonance imaging (MRI). For the retrospective studies (Table 1), sufficient biopsy sample had to be available for detection and quantification of Aβ pathology. All 4 studies were conducted according to the principles of the Declaration of Helsinki and approved by local human ethics boards. Informed consent was obtained from all patients and/or their designated representatives prior to study entry according to local regulations. The number of patients and additional study characteristics are summarized by study in Table 1.

**Table 1:** Number of patients and other study characteristics, by study

	**Study A (Retrospective, US)**	**Study B (Prospective, US)**	**Study C (Retrospective, EU)**	**Study D (Prospective, EU)**
**N**	7	11	15	17
**Hospital setting**	University of Pennsylvania Philadelphia, PA, USA	Johns Hopkins University Baltimore, MD, USA	• University of Eastern Finland Kuopio, Finland	4 neurosurgical units in Finland:
				• Same 2 centers as in Study C
			• Turku University Turku, Finland	• Helsinki University (Helsinki)
				• Seinajoki Central Hospital (Seinajoki)
**Patients**	Retrospectively identified from database.	Patients scheduled for shunt placement were contacted.	Patients had previously undergone ICP monitoring and R frontal brain biopsy for suspected iNPH.	Patients scheduled for shunt placement were contacted.
**Clinical conduct start/end dates**	21 Dec 2009	11 Mar 2010	15 Jun 2010	31 May 2010
	02 Jul 2010	05 Jan 2011	17 Nov 2010	16 Dec 2010
**Order and timing of Biopsy/PET**^**a,b**^	Biopsy/PET, 3 to 45 months apart	PET/Biopsy, approximately 8 weeks apart	Biopsy/PET, 8.8 to 38.2 months apart	PET/Biopsy, approximately 3 weeks apart
**Diagnostic criteria for iNPH**	Patients who had undergone shunting with concomitant R prefrontal cortical biopsy (most patients from a previous study of the impact of AD pathology on clinical response to shunting [18]).	Patients scheduled for shunt placement for iNPH.	Patients had previously undergone 24-hour ICP monitoring and R frontal cortical biopsy.	CT/MRI findings (enlarged ventricles + obliterated cortical sulci) + at least 2 of the 3 cardinal symptoms (abnormal gait, incontinence, cognitive impairment) + if necessary, positive lumbar tap test or 24-hour ICP recording.

AD = Alzheimer’s disease; CT = computed tomography; EU = Europe; ICP = intracranial pressure; iNPH = normal pressure hydrocephalus; MRI = magnetic resonance imaging; PET = positron emission tomography; R = right; US = United States.

^a^In the 2 retrospective studies (n = 22), mean (SD) time between PET and biopsy was 620.91 (384.09) days.

^b^In Study A, a continuous variable regression model included percentage of area of Aβ plaque in biopsy sample tissue as measured by monoclonal antibody to Aβ, 4G8, as dependent variable; the [^18^F]flutemetamol PET standard uptake value ratio (SUVR) for the contralateral volume of interest (VOI) as independent variable; and interval from biopsy to PET scan as covariate. The result was significant for the model, and there was no apparent modulating influence of the interval from biopsy to PET scan (p > 0.21) [20]. The results of the analysis of ipsilateral SUVR VOIs were similar. The same results were achieved using time (in months) as a factor in a similar analysis in Study C (unpublished data).

### Procedures

#### [^18^F]Flutemetamol PET image acquisition, processing, quantitative measures, and methodology precedents

The injected activity, PET imaging equipment used, and details of the reconstruction of the 30-minute summed PET image are shown by study in Table 2.

**Table 2:** **Summary of methods by study: MRI and [**^**18**^**F]flutemetamol PET image acquisition**

	**Study A**	**Study B**	**Study C**	**Study D**
**Injected activity/dose of [**^**18**^**F]flutemetamol (MBq)**	Approximately 185; range, 111 to 197	Mean 200; range, 178 to 252	Mean (SD, 178 (3.4); range, 174 to 185	Mean 176; range, 169 to 184
**MRI image equipment**	3 T Siemens Trio whole-body scanner (Siemens, Erlangen Germany) equipped with a product 8-channel array coil	Siemens 3 T TRIO (Milwaukee, WI, USA)	1.5 T Philips MRI scanner Gyroscan Intera CV Nova Dual system (Philips Medical Systems, Best, The Netherlands)	
**PET imaging equipment**	Allegro whole-body PET scanner (Philips, Andover, MA, USA) obtained in a single FOV	GE Advance PET scanner (GE Medical Systems, Milwaukee, WI, USA) in 3D modes with a 14.875-cm axial FOV obtained in a single FOV	ECAT EXACT HR + scanner (Siemens-CTI, Knoxville, TN, USA)	
**Reconstruction**	Reconstructed by a 3-dimensional row-action maximum likelihood algorithm. Approximate spatial resolution was 6 mm.	Reconstructed to 35 transaxial images of 128 x 128 voxels by a back-projection algorithm using manufacturer-provided software correcting for attenuation, scatter, and dead time. Resolution was approximately 6 mm at full width at half maximum.	Not stated.	Not stated.

FOV = field of view; MRI = magnetic resonance imaging; PET = positron emission tomography.

Note: Information in this table was extracted from the original study publications (refer to text) and supplemented with data from the clinical trial submission documents.

[^18^F]Flutemetamol was manufactured according to Good Manufacturing Process at Cardinal Health, Beltsville, MD for the US sites and Turku PET Center, Turku for the Finland sites, and transported to the sites. At the PET imaging site prior to [^18^F]flutemetamol injection, quality control tests were performed including radioactivity content and chemical purity by high-performance liquid chromatography. The radiopharmacist ensured that the correct activity was present in the injection syringe and that the product was used within the validity period. [^18^F]Flutemetamol (target dose, 185 MBq) was injected over approximately 40 seconds by study staff.

The PET scan was initiated approximately 90 minutes after injection with [^18^F]flutemetamol and lasted 30 minutes (six 5-minute frames). All PET cameras in these studies were qualified with a structured phantom prior to scanning patients in the studies. Each site performed cycles of phantom reconstruction using different filter parameters until the spatial resolution was approximately 6.5 mm (in order to prove approximately equal partial volume effects) [32]. The dynamic PET data was summed over the entire scan to create a 30-minute summation image.

Within 35 days of PET imaging, patients underwent MRI to rule out confounding conditions (e.g., vascular, structural) and facilitate volume of interest (VOI) analysis of [^18^F]flutemetamol retention. The patient’s MRI was co-registered with the [^18^F]flutemetamol PET image. The biopsy site on the [^18^F]flutemetamol image was located from either (1) the post-biopsy MRI scan (for retrospective studies) or (2) an MRI scan co-registered with the post-biopsy computed tomography (CT) scan (for prospective studies).

While the patient’s MRI was used to define the placement of the VOI in the PET image for the biopsy site, workstation functionality enabled location of an equivalent region on the contralateral side. VOIs were manually drawn (1) on the tissue including the biopsy site (prospective studies) or on the tissue surrounding the hollow excised biopsy site (retrospective studies) and (2) on the corresponding contralateral region. For the retrospective studies, measures were taken to minimize the influence (partial volume effect) the prior surgical procedure would have had on measured tracer retention, i.e., slightly larger VOIs were placed around the biopsy site (and matching contralateral site). Mean VOIs are shown in Table 3. A complete discussion of partial volume effect is beyond the scope of this paper, but a review of the topic can be found in [34]. Briefly, radiotracer is retained in white matter in both normal subjects and patients with AD. Tracer signal from white matter may spill over any potential cortical signal in narrow widths of cortical grey matter. In our studies, reference VOIs were placed on 2 sites (cerebellar cortex and pons) to learn whether one reference region (REF) might be preferable to the other.

**Table 3:** **Mean volumes of interest for retrospective and prospective studies**[32]

	**Retrospective studies (Studies A and C)**		**Prospective studies (Studies B and D)**	
	**Mean VOI (SD)**	**Minimum-maximum**	**Mean VOI (SD)**	**Minimum-maximum**
**Ipsilateral**	1.17 mL (0.23)	0.61 – 1.58	0.55 mL (0.07)	0.37 – 0.70
**Contralateral**	0.91 mL (0.25)	0.52 – 1.33	0.54 mL (0.06)	0.42 – 0.71

From studying [^11^C]PiB, which is structurally similar to [^18^F]flutemetamol, we know that (1) regional brain uptake of tracer is proportional to the regional level of brain amyloid, as determined by IHC and HC [22], and (2) symmetrically placed VOIs in the left and right frontal cortices result in similar SUVRs [35].

SUVRs were calculated for the VOIs as follows: SUV_VOI_/SUV_REF_, with SUV being the integrated activity over a given time period per unit of injected dose and body weight. The reference regions, cerebellar cortex and pons, were not expected to have any fibrillar Aβ plaque burden. Previous work has shown that the SUVR range of [^18^F]flutemetamol referenced to cerebellum in normal subjects is between 1.1 and 1.5 [36]. It should be noted, however, that in addition to mature (dense, cored) neuritic plaques, and to a much lesser degree diffuse Aβ plaques, [^18^F]flutemetamol is retained by vascular amyloid deposits [22]. The results from the original studies showed that 3/43 patients examined for vascular amyloid in the pooled dataset were positively identified by the pathologist (1 with an overall pathology diagnosis of normal).

A mean composite cortical VOI was calculated as the mean of several anatomic regions typically associated with significant Aβ plaque burden in AD (frontal cortex, anterior cingulate gyrus, posterior cingulate gyrus/precuneus, lateral-temporal cortex, and parietal cortex). Neocortex is associated with Thal Phase 1 of Aβ deposition in AD, and cingulate is associated with Thal Phase 2 (out of 5 phases) [37]. Thal Phases 1 and 2 correspond to the designation “A1” according to the “ABC” system set forth in the NIA-AA guidelines for the neuropathologic assessment of AD [38]. Similar composite measurements have been previously described for [^11^C]PiB [39], [^18^F]flutemetamol [36], and [^18^F]florbetaben [40]. Since the five brain regions vary considerably in size, and the mean was not corrected for VOI size, the SUVR of the composite VOI reflects the level of uptake as if all 5 regions were of equal importance and not the overall uptake level in the composite VOI. Composite cortical SUVR has resulted in a value (i.e., overall estimate) of the fibrillar Aβ burden in the brain as a whole similar to that for the biopsy VOI [41] and is similar to a global PiB retention summary from 6 cortical regions of interest as justified in [42].

#### [^18^F]Flutemetamol PET blinded image evaluation

Anonymized [^18^F]flutemetamol PET data were transferred to the GE Healthcare Image Review Center in Oslo, Norway, and reviewed according to the studies’ image review charters and in accordance with United States Food and Drug Administration Guidance to Industry [http://www.fda.gov/downloads/Drugs/GuidanceComplianceRegulatoryInformation/Guidances/UCM268555.pdf]. Images were loaded onto Xeleris workstations (GE Medical Systems, Milwaukee, WI) and reviewed using its Volumetrix application.

Visual interpretation of images by readers blinded to subject clinical and pathological information except for iNPH status (blinded image examination [BIE]) was performed by 3 trained, experienced raters (nuclear medicine physicians) in each study, in an individually randomized order of image presentation. Inter-rater agreement has previously been shown to be strong [32]. All readers assessed the grey matter tracer patterns in the same protocol-specified regions, in the same protocol-specified order.

#### Biopsy

Biopsy tissue was taken with biopsy forceps or a 14-gauge biopsy needle. The biopsy sample in Study A was approximately 5 mm^3^ and in the other 3 studies was approximately 14 mm^3^. In Study A, the biopsy was from the right prefrontal cortex; in Studies C and D, right prefrontal cortex, mid-pupillary line in front of the coronal suture was specified. In accordance with neurosurgical procedures approved by the institutional review board for the Johns Hopkins site, parietal cortex was biopsied in Study B.

#### Immunohistochemistry (4G8) and histochemistry (thioflavin S and Bielschowsky silver) methodology and measures

The biopsy tissue was fixed in 10% neutral buffered formalin and embedded in paraffin. If the sample size allowed, up to 50 serial sections were cut at a 5-μm thickness (6 μm in Study A) and numbered sequentially. Wherever possible, 3 sections separated by 100 μm (e.g., Slides 3, 23, and 43) from each biopsy were used for each of 3 staining techniques: 4G8 IHC, Bielschowsky silver, and thioflavin S. 4G8 IHC was standardized using the methodology of Study A as a model, and performed for Studies B, C, and D at Covance Laboratories Ltd (Harrogate, North Yorkshire, UK) as previously detailed [32]. Formic acid pre-treatment was used. Five measures (Aβ percentage area or plaque score, described below) were taken from each slide, and a mean was determined for each specimen.

Automated histometric measurement of the percentage area of Aβ in grey matter in 4G8 stained sections was performed. Except for 4G8 sections from only 7 subjects for whom Aβ percentage area was measured at the University of Pennsylvania using a similar technique (Study A [20]), 4G8 sections were imaged using whole slide scanning (Aperio XT) with a pre-developed and validated macro (MATLAB) used to threshold intensity, size, and morphometry after color deconvolution to remove the hematoxylin staining channel. The 4G8 antibody dilution for the 7 samples from Study A was higher (1:500) than for the remaining 40 available 4G8 samples (1:100). Three of these 7 samples were completely 4G8 negative (0.00% 4G8); 4G8 percentage area was 0.07%, 1.52%, 2.05%, and 2.75%, respectively, in the remaining 4 samples. In Studies B, C, and D a total of 5 out of 40 samples were completely 4G8 negative, and 4G8 percentage area in the remaining 35 samples ranged from 0.01% to 13.41%.

To dichotomise the 4G8 data (normal/abnormal) we used a threshold of 2.5% (receiver-operator characteristics analysis from multiple cortical samples taken in an autopsy study cohort of 68 subjects, data not shown). In 11/47 4G8 samples, the cut-off definition did not match the overall pathology judgment (8 samples with 4G8 positivity ≤2.5% had an overall pathology judgment of abnormal, and 3 samples with 4G8 positivity >2.5% had an overall pathology judgment of normal).

In Bielschowsky silver and thioflavin S stained sections, plaques were counted and scored using the following modified Consortium to Establish a Registry for Alzheimer’s Disease (CERAD) 4-point scale [43]: 0 = no plaques, 1 = sparse plaques (1 to 5), 2 = moderate plaques (6 to 19), and 3 = frequent plaques (20+). For Study A, Bielschowsky silver and thioflavin S plaque counts were not assessed using the same scale as in the other studies, and were therefore not included in the pooled data set. For one subject in Study C, the thioflavin S result (average of 3 sections with plaque scores of 0, 1, and 2, respectively) was changed from abnormal to normal after the results of the study were known. This was justified because, according to the published methods for Study C, only a plaque score of 2 (moderate) or 3 (frequent) for thioflavin S was considered abnormal [30], and justified also according to the standardized analysis definitions for this study. All other outcomes for this subject were abnormal.

All staining methods were pre-validated and optimized prior to study samples being stained. It is acknowledged that Bielschowsky silver stain is not readily scaled up and was performed in small batches along-side control sections. The neuropathologists assessing the sections were at liberty to request re-stains, and comments on the quality of the sections actually assessed were collected. Good, Satisfactory and Poor meant that sections were assessable with varying degrees of ease while the option to record the sections as “Unassessable” was also available.

The physician evaluating the 4G8 slides for Study A was blinded to case identity. The independent neuropathologist at the contract research organization evaluating all other slides was blinded to clinical and imaging data; 4G8 slides for histometric analysis were scanned at a separate location, and the same slides were then shipped and used in the neuropathologic assessment.

### Statistical analysis

All 24 possible combinations (pairs) of the following 4 pathology SoTs and 6 image endpoints were studied:

Pathology data (SoT)

(a) Plaque as a percentage of area IHC-stained with 4G8 antibody

(b) Bielschowsky silver plaque score (normal/abnormal)

(c) Thioflavin S plaque score (normal/abnormal)

(d) Overall pathology (normal/abnormal)

Normal and abnormal are defined below.

Imaging data (SUVR type)

Cerebellum as reference

(i) SUVR for the ipsilateral VOI

(ii) SUVR for the matching contralateral VOI

(iii) SUVR for the composite VOI

Pons as reference

(iv) SUVR for the ipsilateral VOI

(v) SUVR for the matching contralateral VOI

(vi) SUVR for the composite VOI

Additionally, the results of the BIE were an endpoint.

There were only SUVR readings and a BIE reading for 1 patient included in the analysis (no pathology IHC or HC was available for this patient).

An explanation of the sequence and logic of statistical analysis processes is presented at the end of this statistical methodology section, after all the terms have been defined.

Objective 1: The first objective of the statistical analysis was to determine which pair of SUVR/pathology endpoints matched best.

Analysis: For each of the 4 pathology SoTs, the result was dichotomized as normal or abnormal. For 4G8 IHC (a), if the area was ≤2.5% it was classified as normal; otherwise, it was abnormal. For Bielschowsky silver stain (b) and thioflavin S (c), the normal/abnormal plaque score threshold was the midpoint between sparse and moderate (i.e., ≤1.5 was defined as normal). In addition, an overall pathology SoT (d) consisted of the pathologist’s overall impression (normal or abnormal) based on all slides prepared. The overall pathology assessment was a judgment call made by the independent expert neuropathologist (blinded to clinical information) to allow for discrepant results from the 3 staining methods (if any) resulting in the final classification of each case as normal or abnormal.

Based on each pathology SoT, receiver-operator characteristic (ROC) analyses (defined below) were performed, in which the discriminative ability of each SUVR type (i through vi) was tested against each pathology SoT (a through d). For each of these 24 ROC analyses, the area under the curve (AUC) value was determined. In addition, for each ROC curve, the optimal threshold for each SUVR score was determined by the Youden index [44] using MedCalc software (Ostend, Belgium). After obtaining the optimal threshold for each ROC analysis, the sensitivity, specificity, PPV, NPV, and accuracy of each SUVR method for prediction of each SoT were calculated.

Result: The best matched pair (SUVR type and SoT) would be the one which has the highest AUC value in the ROC analysis, the largest sum of sensitivity and specificity [45], or the largest Youden index.

Objective 2: The second objective of the statistical analysis was to determine whether SUVR was a better measure for predicting SoT outcome than the majority BIE result (i.e., at least 2 of 3 readers).

Analysis: Based on each pathology SoT, we compared the sensitivity and specificity of majority BIE read to the sensitivity and specificity of SUVR with the McNemar test (used to compare paired proportions).

Result: The results were expressed as point estimates of the sensitivity and specificity values for both BIE and SUVR, their 95% confidence intervals (CIs), and p-values for the comparisons.

Objective 3: The third objective of the statistical analysis was to determine whether there was a better match between PET imaging findings and pathology findings in retrospective or prospective studies.

Analysis: For each pathology SoT, we determined whether the pairing of PET SUVR type and SoT with the largest sum of sensitivity and specificity was for prospective or retrospective studies. We also determined, for each pathology SoT and PET SUVR type, which study cohort (prospective or retrospective) had the larger sum of sensitivity and specificity.

Result: The results were expressed as point estimates of the sum of the sensitivity and specificity values and their 95% CIs.

#### Definitions and explanation of terms

The scheme for classifying biopsy results is shown in Table 4.

**Table 4:** Scheme for classifying biopsy results

**[**^**18**^**F]flutemetamol PET result**	**Aβ Pathology standard of truth**	
	**Abnormal**	**Normal**
Abnormal	True positive (TP)	False positive (FP)
Normal	False negative (FN)	True negative (TN)

The definitions for diagnostic efficacy are shown in Table 5 where TP, FN, FP, TN are numbers of patients.

**Table 5:** Definitions for diagnostic efficacy of [18F]flutemetamol PET

**Metric**	**Definition**
Sensitivity^a^	TP/TP + FN
Specificity^a^	TN/TN + FP
Accuracy^b^	TP + TN/TP + FN + TN + FP
Positive predicative value (PPV)^a^	TP/TP + FP
Negative predictive value (NPV)^a^	TN/TN + FN

^a^The highest possible value of the result is 1. This result generally does not depend on disease prevalence.

^b^The highest possible accuracy is 1. Accuracy is a function of disease prevalence in the population being tested.

A ROC curve is the graph where the *y* axis represents sensitivity and the *x* axis represents 1 minus specificity. The ROC AUC is a measure of test performance with a ROC AUC of 1 indicating a perfect test.

The sum of sensitivity and specificity indicates whether a diagnostic test will result in a revision of the pre-test probability of disease [45]. The highest possible sum of sensitivity and specificity is 2.

The Youden index is the sum of sensitivity and specificity minus 1; a perfect test would have a Youden index of 1.

#### SUVR thresholds

Using all of the available SUVR values for each combination of the 6 SUVR types, an estimate was made of the SUVR value that would maximize the value of the Youden index. These maximal Youden values and their optimal SUVR values are shown in Table 6. These SUVR values that maximized the Youden index values were then used to calculate ROC AUCs (also shown in Table 6).

**Table 6:** ROC AUC, Youden index, and Optimal SUVR thresholds by SUVR type and pathology SoT for all 4 studies combined

**Pathology standard of truth**				
**SUVR type**				
**SUVR brain region**	**REF**	**ROC AUC**	**Maximal Youden index**	**SUVR cut-off criterion**
4G8				
Ipsilateral	Cerebellum	0.8544	0.6216	>1.25
Contralateral	Cerebellum	0.8529	0.6757	>1.24
Composite	Cerebellum	0.7508	0.5315	>1.79
Ipsilateral	Pons	0.7763	0.5000	>0.42
Contralateral	Pons	0.7853	0.5468	>0.44
Composite	Pons	0.7583	0.5205	>0.45
Bielschowsky				
Ipsilateral	Cerebellum	0.9769	0.8889	>1.31
Contralateral	Cerebellum	0.9769	0.8889	>1.31
Composite	Cerebellum	0.9815	0.9259	>1.38
Ipsilateral	Pons	0.9699	0.8214	>0.46
Contralateral	Pons	0.9815	0.9286	>0.48
Composite	Pons	0.9792	0.8571	>0.48
Thioflavin S				
Ipsilateral	Cerebellum	0.9288	0.7465	>1.62
Contralateral	Cerebellum	0.9253	0.7951	>1.31
Composite	Cerebellum	0.9462	0.8438	>1.34
Ipsilateral	Pons	0.8767	0.6465	>0.46
Contralateral	Pons	0.9132	0.7980	>0.48
Composite	Pons	0.9236	0.7374	>0.5
Overall pathology				
Ipsilateral	Cerebellum	0.9916	0.9286	>1.5
Contralateral	Cerebellum	0.9979	0.9706	>1.31
Composite	Cerebellum	1.0000	1.0000	>1.46
Ipsilateral	Pons	0.9433	0.7857	>0.472
Contralateral	Pons	0.9842	0.8857	>0.479
Composite	Pons	0.9947	0.9286	>0.53

AUC = area under the curve; REF = brain reference region; ROC = receiver-operator curve; SUVR = standard uptake value ratio; SoT = standard of truth; VOI = volume of interest.

Note: SUVR cut-off criterion is the SUVR value that maximizes the value of the Youden index. The SUVR cut-off value is the SUVR value above which lies a reading of abnormal and below or equal to which is a reading of normal.

#### Sequence and logic of statistical analysis process

Here is the sequence and logic for the statistical analysis process. There are 24 combinations of SUVR type (6) and pathology SoT (4). For each of these 24 combinations there are approximately 49 sets of SUVR values (quantitative—continuous scale) and pathology SoT value (always dichotomized—normal/abnormal as described above). For each of 24 combinations, an ROC curve is generated using the “population” of data (SUVR and SoT values). For any 1 SUVR value, a point on the ROC is determined, since both sensitivity and specificity are determined by use of that SUVR value as a cut-off (SUVR values below that cut-off are termed normal, and SUVR values above that cut-off are termed abnormal; each SoT value is already labeled as normal or abnormal by the pathology cut-off values).

Considering all values on the ROC curve, 1 value on the ROC curve will be a maximum value for the Youden index, which is just (sensitivity + specificity -1). If the Youden index is maximized at a point on the ROC curve, then clearly the sum of sensitivity and specificity will also be maximal at the same point on the ROC curve. We recorded at what SUVR cut-off value the maximal Youden index (and thus the maximal sum of sensitivity and specificity) was found. These are the so-called “optimal SUVR thresholds” (Table 6 in Results). All subsequent diagnostic efficacy parameters were computed using this optimal SUVR threshold. Thus, in Table 7, the “sum of sensitivity and specificity values” are actually the “maximal sum of sensitivity and specificity values” for each combination of SUVR type and pathology SoT. The sensitivity and specificity values given for each combination are those at the SUVR threshold that maximized the sum of sensitivity and specificity for that combination.

**Table 7:** Sensitivity and specificity and their exact 95% CIs for each SUVR type/pathology SoT combination for all 4 studies combined

	**Cerebellum REF**			**Pons REF**		
**Pathology SoT**	**Ipsilateral**	**Contralateral**	**Composite**	**Ipsilateral**	**Contralateral**	**Composite**
**4G8**						
Sensitivity (95% CI)	1.0000 (0.6637, 1.0000)	1.0000 (0.6637, 1.0000)	0.6667 (0.2993, 0.9251)	1.0000 (0.6637, 1.0000)	0.8889 (0.5175, 0.9972)	0.8889 (0.5175, 0.9972)
Specificity (95% CI)	0.6216 (0.4476, 0.7754)	0.6757 (0.5021, 0.8199)	0.8649 (0.7123, 0.9546)	0.5000 (0.3338, 0.6662)	0.6579 (0.4865, 0.8037)	0.6316 (0.4599, 0.7819)
Maximal sum (sensitivity + specificity)	1.6216	1.6757	1.5316	1.5000	1.5468	1.5205
**Bielschowsky**						
Sensitivity (95% CI)	1.0000 (0.6306, 1.0000)	1.0000 (0.6306, 1.0000)	1.0000 (0.6306, 1.0000)	1.0000 (0.6306, 1.0000)	1.0000 (0.6306, 1.0000)	1.0000 (0.6306, 1.0000)
Specificity (95% CI)	0.8889 (0.7084, 0.9765)	0.8889 (0.7084, 0.9765)	0.9259 (0.7571, 0.9909)	0.8214 (0.6311, 0.9394)	0.9286 (0.7650, 0.9912)	0.8571 (0.6733, 0.9597)
Maximal sum (sensitivity + specificity)	1.8889	1.8889	1.9259	1.8214	1.9286	1.8571
**Thioflavin S**						
Sensitivity (95% CI)	0.7778 (0.3999, 0.9719)	0.8889 (0.5175, 0.9972)	1.0000 (0.6637, 1.0000)	0.8889 (0.5175, 0.9972)	0.8889 (0.5175, 0.9972)	0.8889 (0.5175, 0.9972)
Specificity (95% CI)	0.9688 (0.8378, 0.9992)	0.9063 (0.7498, 0.9802)	0.8438 (0.6721, 0.9472)	0.7576 (0.5774, 0.8891)	0.9091 (0.7567, 0.9808)	0.8485 (0.6810, 0.9489)
Maximal sum (sensitivity + specificity)	1.7466	1.7952	1.8438	1.6465	1.7980	1.7374
**Overall pathology**						
Sensitivity (95% CI)	0.9286 (0.6613, 0.9982)	1.0000 (0.7684, 1.0000)	1.0000 (0.7684, 1.0000)	0.9286 (0.6613, 0.9982)	1.0000 (0.7684, 1.0000)	0.9286 (0.6613, 0.9982)
Specificity (95% CI)	1.0000 (0.8972, 1.0000)	0.9706 (0.8467, 0.9993)	1.0000 (0.8972, 1.0000)	0.8571 (0.6974, 0.9519)	0.8857 (0.7326, 0.9680)	1.0000 (0.9000, 1.0000)
Maximal sum (sensitivity + specificity)	1.9286	1.9706	2.0000	1.7857	1.8857	1.9286

CI = confidence interval; REF = PET imaging brain reference region; SoT = standard of truth, SUVR = standard uptake value ratio.

The number and percentage of TP, FN, TN, and FP values (refer to Table 4) given in Table 8 (and the values computed from them (accuracy, PPV, and NPV) are all values that have been determined at the optimal SUVR threshold, that is, the SUVR threshold that maximized the Youden index/sum of sensitivity and specificity for that combination of SUVR type and pathology SoT.

**Table 8:** **Accuracy of [**^**18**^**F]flutemetamol quantitative diagnosis and positive and negative predictive values by Aβ pathology SoT in the 4 studies combined**

**Pathology standard of truth**									
**SUVR type**		**SUVR/SoT, N (%)**				**N**	**(%)**		
**Brain SUVR region**	**REF**	**TN n (%)**	**FN n (%)**	**FP n (%)**	**TP n (%)**	**Total**	**Accuracy**^**a**^	**PPV**^**b**^	**NPV**^**c**^
4G8% area									
Ipsi	C	23 (62)	0 (0)	14 (38)	9 (100)	46	69.57	39.13	100.00
Contra	C	25 (68)	0 (0)	12 (32)	9 (100)	46	73.91	42.86	100.00
Comp	C	32 (86)	3 (33)	5 (14)	6 (67)	46	82.61	54.55	91.43
Ipsi	P	19 (50)	0 (0)	19 (50)	9 (100)	47	59.57	32.14	100.00
Contra	P	25 (66)	1 (11)	13 (34)	8 (89)	47	70.21	38.10	96.15
Comp	P	24 (63)	1 (11)	14 (37)	8 (89)	47	68.09	36.36	96.00
Bielschowsky silver plaque score									
Ipsi	C	24 (89)	0 (0)	3 (11)	8 (100)	35	91.43	72.73	100.00
Contra	C	24 (89)	0 (0)	3 (11)	8 (100)	35	91.43	72.73	100.00
Comp	C	25 (93)	0 (0)	2 (7)	8 (100)	35	94.29	80.00	100.00
Ipsi	P	23 (82)	0 (0)	5 (18)	8 (100)	36	86.11	61.54	100.00
Contra	P	26 (93)	0 (0)	2 (7)	8 (100)	36	94.44	80.00	100.00
Comp	P	24 (86)	0 (0)	4 (14)	8 (100)	36	88.89	66.67	100.00
Thioflavin S plaque score									
Ipsi	C	31 (97)	2 (22)	1 (3)	7 (78)	41	92.68	87.50	93.94
Contra	C	29 (91)	1 (11)	3 (9)	8 (89)	41	90.24	72.73	96.67
Comp	C	27 (84)	0 (0)	5 (16)	9 (100)	41	87.80	64.29	100.00
Ipsi	P	25 (76)	1 (11)	8 (24)	8 (89)	42	78.57	50.00	96.15
Contra	P	30 (91)	1 (11)	3 (9)	8 (89)	42	90.48	72.73	96.77
Comp	P	28 (85)	1 (11)	5 (15)	8 (89)	42	85.71	61.54	96.55
Overall pathology									
Ipsi	C	34 (100)	1 (7)	0 (0)	13 (93)	48	97.92	100.00	97.14
Contra	C	33 (97)	0 (0)	1 (3)	14 (100)	48	97.92	93.33	100.00
Comp	C	34 (100)	0 (0)	0 (0)	14 (100)	48	100.00	100.00	100.00
Ipsi	P	30 (86)	1 (7)	5 (14)	13 (93)	49	87.76	72.22	96.77
Contra	P	31 (89)	0 (0)	4 (11)	14 (100)	49	91.84	77.78	100.00
Comp	P	35 (100)	1 (7)	0 (0)	13 (93)	49	97.96	100.00	97.22

C = cerebellum; Comp = composite; Contra = contralateral; FN = false negatives; FP = false positives; Ipsi = ipsilateral; N = number of patients; NPV = negative predictive value; P = pons; PPV = positive predictive value; REF = PET imaging brain reference region; ROC = receiver-operator curve; SoT = standard of truth; TN = true negatives; TP = true positives; SUVR = standard uptake value ratio.

It is not shocking that the larger ROC AUCs generally occur with larger Youden indices, since a large Youden index means a large sum of sensitivity and specificity, and the maximal ROC AUC is observed when both sensitivity and specificity are 1.0. The Sigma Plot instructions for ROC curve analysis state “An important measure of the accuracy of the clinical test is the area under the ROC curve. If this area is equal to 1.0 then the ROC curve consists of two straight lines, one vertical from 0,0 to 0,1 and the next horizontal from 0,1 to 1,1. This test is 100% accurate because both the sensitivity and specificity are 1.0 so there are no false positives and no false negatives.”

Comparing diagnostic efficacy values using BIE and pathology SoT is essentially exactly the same as using dichotomized SUVR values. In both cases, the imaging judgment has been dichotomized into normal or abnormal, either from using a cut-off value for SUVR or an immediate visual judgment for BIE.

## Results

**Figure 1:**
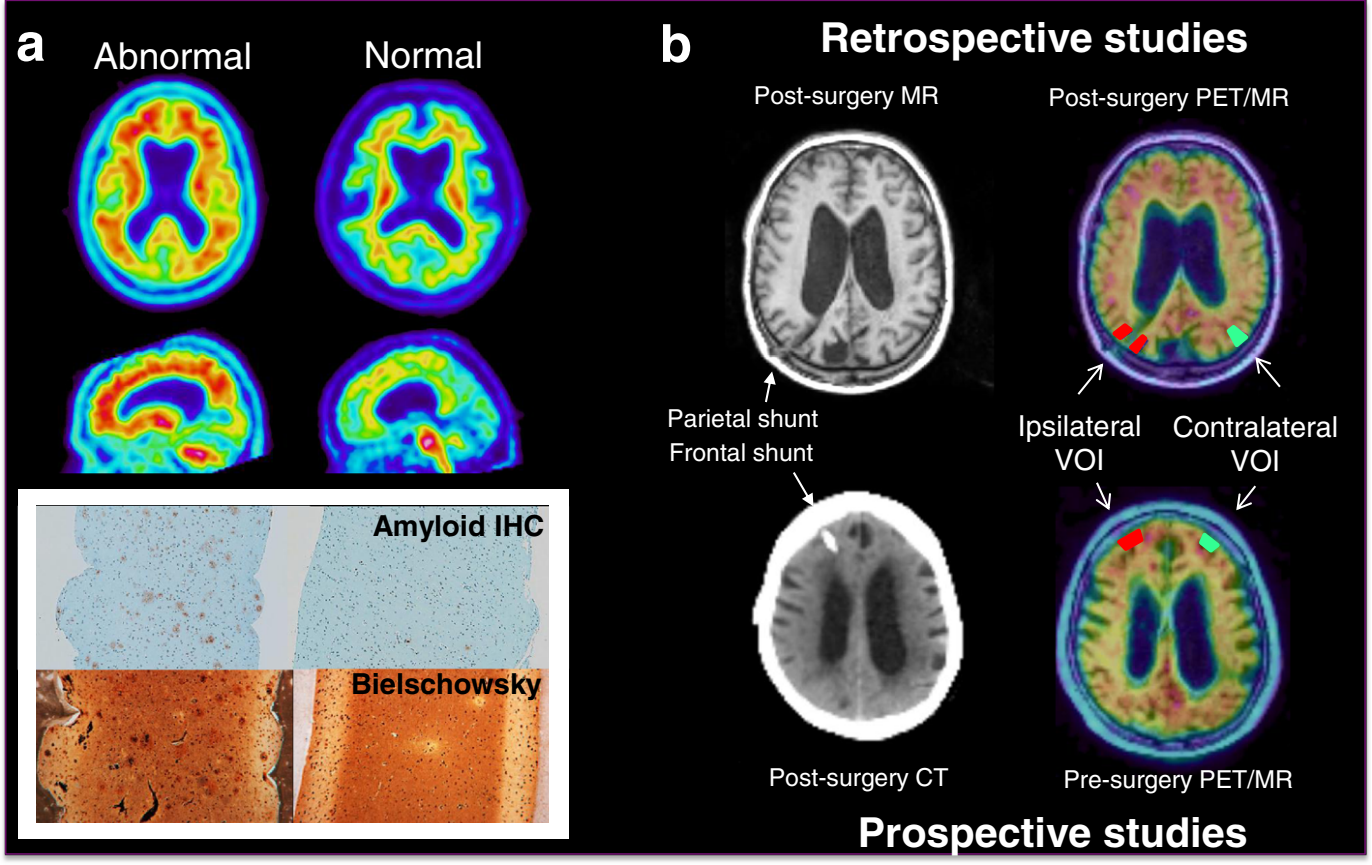
**Examples of abnormal and normal [**^**18**^**F]flutemetamol positron emission tomography (PET) and corresponding magnetic resonance (MR) or computed tomography (CT) imaging and histopathology.** Panel **a**) [^18^F]Flutemetamol PET imaging correlates with histopathology (Study D). Amyloid plaques were determined in biopsy samples by 4G8 imunohistochemistry (IHC). Neuritic plaques were identified in serial sections using a modified Bielschowsky silver stain. Panel **b**) [^18^F]Flutemetamol PET images were obtained either retrospectively after biopsy (Studies A and C) or prospectively before biopsy (Studies B and D). Small cortical biopsies were taken during shunt placement and histopathology was correlated to standard uptake value ratio (SUVR) measures in volumes of interest (VOIs) either ipsilateral or contralateral to the site of biopsy.

Unless stated otherwise, results shown are for Studies A, B, C, and D combined. Representative photomicrographs of Bielschowsky silver stain and 4G8 stain are displayed in Figure 1 alongside examples of abnormal and normal PET images.

### SUVR type/pathology SoT pairs with highest ROC AUC and largest Youden index

Including all data from the 4 pooled studies combined, for 3 of the 4 pathology SoTs, the SUVR type with the largest ROC AUC was the composite SUVR referenced to the cerebellum (composite-cerebellum) (overall pathology [AUC = 1.0000], Bielschowsky silver [AUC = 0.9815], and thioflavin S [AUC = 0.9462]) (Table 6). For Bielschowsky silver, the ROC AUC was as large for contralateral-cerebellum as for composite-cerebellum. For the fourth pathology SoT, the SUVR type with the largest ROC AUC completing the pair was ipsilateral-cerebellum (4G8 [AUC = 0.8544]).

**Figure 2:**
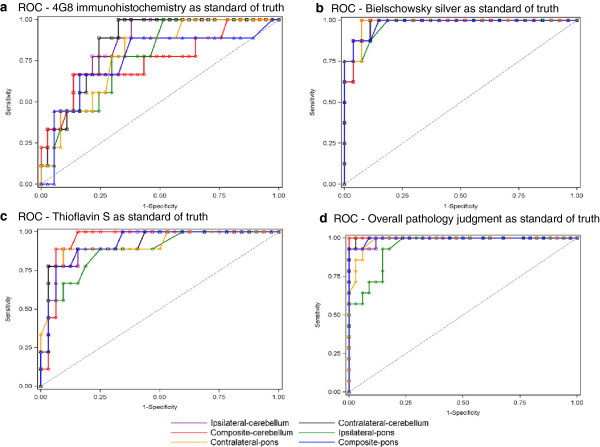
**Receiver-operator curves by pathology standard of truth for each SUVR type: a) 4G8, b) Bielschowsky silver stain, c) Thioflavin S, and d) Overall Pathology.** The composite-cerebellum/overall pathology pair had the largest ROC AUC (1.000). ROC AUCs for composite-cerebellum/and contralateral-pons/Bielschowsky silver were nearly as large (both 0.9815).

Considering all SUVR type/pathology SoT pairs, the composite-cerebellum/overall pathology pair had the largest ROC AUC (1.000). ROC AUCs for composite-cerebellum/and contralateral-pons/Bielschowsky silver were nearly as large (both 0.9815) (Figure 2).

The combination of SUVR type/pathology SoT with the largest Youden index (1.0000) was composite-cerebellum/overall pathology (Table 6). For the other 3 pathology SoTs, the SUVR types with the largest Youden index in descending order were as follows: contralateral-pons/Bielschowsky silver (0.9286), composite-cerebellum/thioflavin S (0.8438), and contralateral-cerebellum/4G8 (0.6757).

The largest Youden index for each pathology SoT was almost always found in the SUVR type/SoT pair with the largest ROC AUC. The exception was for 4G8, with the largest Youden index for the combination with ROC AUC of 0.8529, not the combination with the ROC AUC of 0.8544 (Table 6).

All of the SUVR cut-off criteria using cerebellum as the reference region were greater than 1. None of the SUVR cut-off criteria using pons as the reference region were greater than 1.

### SUVR type/pathology SoT pairs with largest sum of sensitivity and specificity (4 studies combined) (Table 7)

The SUVR type with the highest sum of sensitivity and specificity for each SoT in order of descending value was composite-cerebellum/overall pathology (2.000), contralateral-pons/Bielschowsky silver (1.9286), composite-cerebellum/thioflavin S (1.8438), and contralateral-cerebellum/4G8 (1.6757).

For 4G8, sensitivity was good, greater than 0.8889 for all SUVR types (and similar to that for the other pathology SoTs), but specificity was poorer (range, 0.5000 to 0.8649).

**Figure 3:**
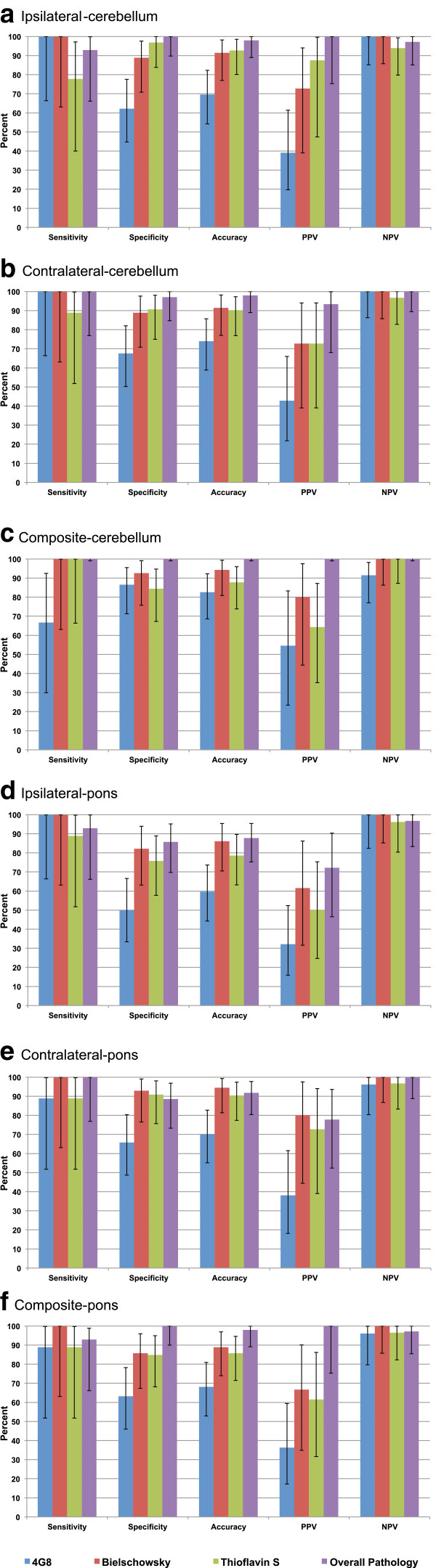
**Diagnostic efficacy by SUVR type (a – c using cerebellum as reference region, d – f using pons as reference region) for each pathology standard of truth (within each group from left to right: 4G8 [blue], Bielschowsky Silver [rust], Thioflavin S [green], and Overall Pathology [purple]).** Horizontal axis: Groups of bars from left to right represent Sensitivity, Specificity, Accuracy, PPV, and NPV. Vertical axis: Percentage (maximum 100%). Error bars represent 95% confidence intervals.

Sensitivity, specificity, accuracy, PPV, and NPV values are illustrated in bar graphs for each pathology SoT in Figure 3.

### Image reading method/pathology SoT pairs with largest sum of sensitivity and specificity (quantitative SUVR vs. BIE) (Table 9)

**Table 9:** Sensitivity, specificity, and exact 95% CIs for each image reading method (quantitative vs. majority BIE) by pathology SoT for all 4 studies combined

	**Cerebellum REF**			**Pons REF**		
**Pathology SoT**	**Ipsilateral**	**Contralateral**	**Composite**	**Ipsilateral**	**Contralateral**	**Composite**
***4G8***						
**Quantitative (SUVR)**						
Sensitivity (95% CI)	1.0000 (0.6637, 1.0000)	1.0000 (0.6637, 1.0000)	0.6667 (0.2993, 0.9251)	1.0000 (0.6637, 1.0000)	0.8889 (0.5175, 0.9972)	0.8889 (0.5175, 0.9972)
Specificity (95% CI)	0.6216* (0.4476, 0.7754)	0.6757 (0.5021, 0.8199)	0.8649 (0.7123, 0.9546)	0.5000* (0.3338, 0.6662)	0.6579* (0.4865, 0.8037)	0.6316 ^a^ (0.4599, 0.7819)
Sum (sensitivity + specificity)	1.6216	1.6757	1.5316	1.5000	1.5468	1.5205
**BIE**						
Sensitivity (95% CI)	0.6667 (0.2993, 0.9251)	0.6667 (0.2993, 0.9251)	0.6667 (0.2993, 0.9251)	0.6667 (0.2993, 0.9251)	0.6667 (0.2993, 0.9251)	0.6667 (0.2993, 0.9251)
Specificity (95% CI)	0.8108* (0.6484, 0.9204)	0.8108 (0.6484, 0.9204)	0.8108 (0.6484, 0.9204)	0.8158* (0.6567, 0.9226)	0.8158* (0.6567, 0.9226)	0.8158* (0.6567, 0.9226)
Sum (sensitivity + specificity)	1.4775	1.4825	1.4775	1.4825	1.4825	1.4825
***Bielschowsky***						
**Quantitative (SUVR)**						
Sensitivity (95% CI)	1.0000 (0.6306, 1.0000)	1.0000 (0.6306, 1.0000)	1.0000 (0.6306, 1.0000)	1.0000 (0.6306, 1.0000)	1.0000 (0.6306, 1.0000)	1.0000 (0.6306, 1.0000)
Specificity (95% CI)	0.8889 (0.7084, 0.9765)	0.8889 (0.7084, 0.9765)	0.9259 (0.7571, 0.9909)	0.8214 (6311, 9394)	0.9286 (0.7650, 0.9912)	0.8571 (0.6733, 0.9597)
Sum (sensitivity + specificity)	1.8889	1.8889	1.9259	1.8214	1.9286	1.8571
**BIE**						
Sensitivity (95% CI)	1.0000 (0.6306, 1.0000)	1.0000 (0.6306, 1.0000)	1.0000 (0.6306, 1.0000)	1.0000 (0.6306, 1.0000)	1.0000 (0.6306, 1.0000)	1.0000 (0.6306, 1.0000)
Specificity (95% CI)	0.9630 (0.8103, 0.9991)	0.9630 (0.8103, 0.9991)	0.9630 (0.8103, 0.9991)	0.9643 (0.9165, 0.9991)	0.9643 (0.8165, 0.9991)	0.9643 (0.8165, 0.9991)
Sum (sensitivity + specificity)	1.9630	1.9630	1.9630	1.9643	1.9643	1.9643
***Thioflavin S***						
**Quantitative (SUVR)**						
Sensitivity (95% CI)	0.7778 (0.3999, 0.9719)	0.8889 (0.5175, 0.9972)	1.0000 (0.6637, 1.0000)	0.8889 (0.5175, 0.9972)	0.8889 (0.5175, 0.9972)	0.8889 (0.5175, 0.9972)
Specificity (95% CI)	0.9688 (0.8378, 0.9992)	0.9063 (0.7498, 0.9802)	0.8438 (0.6721, 0.9472)	0.7576* (0.5774, 0.8891)	0.9091 (0.7567, 0.9808)	0.8485 (0.6810, 0.9489)
Sum (sensitivity + specificity)	1.7466	1.7952	1.8438	1.6465	1.7980	1.7374
**BIE**						
Sensitivity (95% CI)	0.7778 (0.3999, 0.9719)	0.7778 (0.3999, 0.9719)	0.7778 (0.3999, 0.9719)	0.7778 (0.3999, 0.9719)	0.7778 (0.3999, 0.9719)	0.7778 (0.3999, 0.9719)
Specificity (95% CI)	0.9375 (0.7919, 0.9923)	0.9375 (0.7919, 0.9923)	0.9375 (0.7919, 0.9923)	0.9394* (0.7977, 0.9926)	0.9394 (0.7977, 0.9926)	0.9394 (0.7977, 0.9926)
Sum (sensitivity + specificity)	1.7153	1.7153	1.7153	1.7172	1.7172	1.7172
***Overall pathology***						
**Quantitative (SUVR)**						
Sensitivity (95% CI)	0.9286 (0.6613, 0.9982)	1.0000 (0.7684, 1.0000)	1.0000 (0.7684, 1.0000)	0.9286 (0.6613, 0.9982)	1.0000 (0.7684, 1.0000)	0.9286 (0.6613, 0.9982)
Specificity (95% CI)	1.0000 (0.8972, 0.1.000)	0.9706 (0.8467, 0.9993)	1.0000 (0.8972, 1.0000)	0.8571 (0.6974, 0.9519)	0.8857 (0.7326, 0.9680)	1.0000 (0.9000, 1.0000)
Sum (sensitivity + specificity)	1.9286	1.9706	2.000	1.7857	1.8857	1.9286
**BIE**						
Sensitivity (95% CI)	0.9286 (0.6613, 0.9982)	0.9286 (0.6613, 0.9982)	0.9286 (0.6613, 0.9982)	0.9286 (0.6613, 0.9982)	0.9286 (0.6613, 0.9982)	0.9286 (0.6613, 0.9982)
Specificity (95% CI)	1.0000 (0.8972, 1.0000)	1.0000 (0.8972, 1.0000)	1.0000 (0.8972, 1.0000)	1.0000 (0.9000, 1.0000)	1.0000 (0.9000, 1.0000)	1.0000 (0.9000, 1.0000)
Sum (sensitivity + specificity)	1.9286	1.9286	1.9286	1.9286	1.9286	1.9286

BIE = blinded image examination majority read; CI = confidence interval; REF = PET imaging brain reference region; SoT = standard of truth, SUVR = standard uptake value ratio. Refer to [32] for a discussion of agreement between readers.

*Specificity was higher for BIE than for quantitative SUVR (p <0.05).

When using Bielschowsky silver as the SoT, the sum of sensitivity and specificity was greater for BIE than for the quantitative SUVR for all of the SUVR types (6/6) (range of differences across SUVR types, 0.0357 [3.57%] to 0.1429 [14.29%]).

When using 4G8 or thioflavin S as the SoT, the sum of sensitivity and specificity was greater for quantitative SUVR than for the BIE for the majority of the SUVR types (6/6 for 4G8, 5/6 for thioflavin S) (Table 9). The only statistically significant differences were that specificity for BIE majority read was higher than specificity for the quantitative SUVR for the following: for 4G8, ipsilateral-cerebellum and all SUVR types-pons; and for thioflavin S, ipsilateral-pons.

Using overall pathology as the SoT, the sum of sensitivity and specificity for BIE and quantitative SUVR were tied (BIE was higher for 2 of 6 SUVR types, quantitative SUVR was higher for 2 of 6 SUVR types, and BIE and quantitative SUVR were tied for 2 of 6 SUVR types).

### SUVR type/pathology SoT pairs with largest sum of sensitivity and specificity (retrospective vs. prospective studies) (Table 10)

**Table 10:** Sensitivity, specificity, and exact 95% CI for each SUVR type/pathology SoT combination in retrospective vs. prospective studies for all 4 studies combined

	**Cerebellum REF**			**Pons REF**		
**Pathology SoT**	**Ipsilateral**	**Contralateral**	**Composite**	**Ipsilateral**	**Contralateral**	**Composite**
***4G8***						
**Retrospective**						
Sensitivity (95% CI)	1.0000 (0.3976, 1.0000)	1.0000 (0.3976, 1.0000)	0.5000 (0.0676, 0.9324)	1.0000 (0.3976, 1.0000)	0.7500 (0.1941, 0.9937)	0.7500 (0.1941, 0.9937)
Specificity (95% CI)	0.6000 (0.3605, 0.8088)	0.7500 (0.5090, 0.9134)	0.9000 (0.6830, 0.9877)	0.4762 (0.2571, 0.7022)	0.6667 ()0.4303, 0.8541	0.6667 (0.4303, 0.8541)
Sum (sensitivity + specificity)	1.6000	1.7500	1.4000	1.4762	1.4167	1.4167
**Prospective**						
Sensitivity (95% CI)	1.0000 (0.4782, 1.0000)	1.0000 (0.4782, 1.0000)	0.8000 (0.2836, 0.9949)	1.0000 (0.4782, 1.0000)	1.0000 (0.4782, 1.0000)	1.0000 (0.4782, 1.0000)
Specificity (95% CI)	0.6471 (0.3833, 0.8579)	0.5882 (0.3292, 0.8156)	0.8235 (0.5657, 0.9620)	0.5294 (0.2781, 0.7702)	0.6471 (0.3833, 0.8579)	0.5882 (0.3292, 0.8156)
Sum (sensitivity + specificity)	1.6471	1.5882	1.6235	1.5294	1.6471	1.5882
***Bielschowsky***						
**Retrospective**						
Sensitivity (95% CI)	1.0000 (0.4782, 1.0000)	1.0000 (0.4782, 1.0000)	1.0000 (0.4782, 1.0000)	1.0000 (0.4782, 1.0000)	1.0000 (0.4782, 1.0000)	1.0000 (0.4782, 1.0000)
Specificity (95% CI)	0.8750 (0.6165, 0.9845)	0.8750 (0.6165, 0.9845)	0.9375 (0.6977, 0.9984)	0.7647 (0.5010, 0.9319)	0.8824 (0.6356, 0.9854)	0.8824 (0.6356, 0.9854)
Sum (sensitivity + specificity)	1.8750	1.8750	1.9375	1.7647	1.8824	1.8824
**Prospective**						
Sensitivity (95% CI)	1.0000 (0.2924, 1.0000)	1.0000 (0.2924, 1.0000)	1.0000 (0.2924, 1.0000)	1.0000 (0.2924, 1.0000)	1.0000 (0.2924, 1.0000)	1.0000 (0.2924, 1.0000)
Specificity (95% CI)	0.9091 (0.5872, 0.9977)	0.9091 (0.5872, 0.9977)	0.9091 (0.5872, 0.9977)	0.9091 (0.5872, 0.9977)	1.0000 (0.7151, 1.000)	0.8182 (0.4822, 0.9772)
Sum (sensitivity + specificity)	1.9091	1.9091	1.9091	1.9091	2.0000	1.8182
***Thioflavin S***						
**Retrospective**						
Sensitivity (95% CI)	0.8571 (0.4213, 0.9964)	0.8571 (0.4213, 0.9964)	1.0000 (0.5904, 1.0000)	0.8571 (0.4213, 0.9964)	0.8571 (0.4213, 0.9964)	0.8571 (0.4213, 0.9964)
Specificity (95% CI)	1.0000 (0.8235, 1.0000)	0.9474 (0.7397, 0.9987)	0.8947 (0.6686, 0.9870)	0.7000 (0.4572, 0.8811)	0.9000 (0.6830, 0.9877)	0.9000 (0.6830, 0.9877)
Sum (sensitivity + specificity)	1.8571	1.8045	1.8947	1.5571	1.7571	1.7571
**Prospective**						
Sensitivity (95% CI)	0.5000 (0.0126, 0.9874)	1.0000 (0.1581, 1.0000)	1.0000 (0.1581, 1.0000)	1.0000 (0.1581, 1.0000)	1.0000 (0.1581, 1.0000)	1.0000 (0.1581, 1.0000)
Specificity (95% CI)	0.9231 (0.6397, 0.9981)	0.8462 (0.5455, 0.9808)	0.7692 (0.4619, 0.9496)	0.8462 (0.5455, 0.9808)	0.9231 (0.6397, 0.9981)	0.7692 (0.4619, 0.9496)
Sum (sensitivity + specificity)	1.4231	1.8462	1.7692	1.8462	1.9231	1.7692
***Overall pathology***						
**Retrospective**						
Sensitivity (95% CI)	1.0000 (0.5407, 1.0000)	1.0000 (0.5407, 1.0000)	1.0000 (0.5407, 1.0000)	1.0000 (0.5407, 1.0000)	1.0000 (0.5407, 1.0000)	1.0000 (0.5407, 1.0000)
Specificity (95% CI)	1.0000 (0.8316, 1.000)	0.9500 (0.7513, 9987)	1.0000 (0.8316, 1.0000)	0.8095 (0.5809, 0.9455)	0.8571 (0.6366, 0.9695)	1.0000 (0.8389, 1.000)
Sum (sensitivity + specificity)	2.0000	1.9500	2.0000	1.8095	1.8571	2.0000
**Prospective**						
Sensitivity (95% CI)	0.8750 (0.4735, 0.9968)	1.0000 (0.6306, 1.0000)	1.0000 (0.6306, 1.0000)	0.8750 (0.4735, 0.9968)	1.0000 (0.6306, 1.0000)	0.8750 (0.4735, 0.9968)
Specificity (95% CI)	1.0000 (0.7684, 1.000)	1.0000 (0.7684, 1.0000)	1.0000 (0.7684, 1.0000)	0.9286 (0.6613, 0.9982)	0.9286 (0.6613, 0.9982)	1.0000 (0.7684, 1.0000)
Sum (sensitivity + specificity)	1.8750	2.0000	2.0000	1.8036	1.9286	1.8750

REF = PET imaging brain reference region; SoT = standard of truth, SUVR = standard uptake value ratio. Refer to [32] for a discussion of agreement between readers.

For the pathology SoTs Bielschowsky silver, thioflavin S, and 4G8, in the majority of SUVR type/pathology SoT combinations (5/6, 4/6, and 4/6, respectively), the sum of sensitivity and specificity was larger for prospective rather than retrospective studies (Table 10).

For the overall pathology SoT, SUVR type/pathology SoT the sum of sensitivity and specificity was tied in 1 of 6 combination pairs, larger in retrospective studies in 3 of 6 pairs, and larger in prospective studies in 2 of 6 pairs.

For retrospective studies, the SUVR types with the highest sum of sensitivity and specificity by pathology SoT in descending order were as follows: for overall pathology, ipsilateral-cerebellum, composite-cerebellum, and composite-pons (2.00, all 3 pairs); for Bielschowsky silver, composite-cerebellum (1.9375); for thioflavin S, composite-cerebellum (1.8947); and for 4G8, contralateral-cerebellum (1.75).

For prospective studies, the SUVR types with the highest sum of sensitivity and specificity by pathology SoT in descending order were as follows: for overall pathology, contralateral-cerebellum and composite-cerebellum (both 2.00); for Bielschowsky silver, contralateral-pons (2.00); for thioflavin S, contralateral-pons (1.9231); and for 4G8, ipsilateral-cerebellum and contralateral-pons (both 1.6471).

### Accuracy of quantitative SUVR diagnosis, positive and negative predictive values

In addition to sensitivity and specificity, accuracy, PPV, and NPV were calculated (Table 8). The numbers and proportions of FN and FP results were lowest in the overall pathology SoT. For each of the 3 stains (IHC and HC), the proportions of patients with FP are substantially greater than the proportions of patients with FN.

SUVR types that had a 100% PPV were only found in the overall pathology SoT (ipsilateral-cerebellum, composite-cerebellum, and composite-pons). All SUVR types had a 100% NPV for the Bielschowsky silver SoT. Composite-cerebellum had 100% accuracy for 3 of the 4 SoTs (all but 4G8). The 4G8 pathology SoT had the lowest PPV and accuracy.

## Discussion

Given that the SUVR of the composite VOI does not reflect the overall uptake level in the composite VOI (i.e., the mean value is not corrected for VOI size and all regions are treated as though they were of equal importance) it is perhaps unexpected that the composite measure for SUVR referenced to the cerebellum for all 4 studies combined was a better match with biopsy pathology findings for 2 pathology SoTs (overall pathology and Bielschowsky silver) than was the SUVR (any combination) from the biopsy region itself or its contralateral homolog. However, when the retrospective and prospective studies were analyzed separately, for retrospective studies, composite-cerebellum was generally the best match for the SoTs, while for the prospective studies, contralateral-pons was generally the best match for the SoTs. This could have been due to the general study limitation that there was a clear cut difference between the retrospective and prospective studies in the size of the VOI assessed. We acknowledge the general limitation that our data were not analyzed without the partial volume correction.

The largest ROC AUC for the combination of SUVR type and pathology SoT (4 studies combined) was for composite-cerebellum SUVR/overall pathology SoT, the most inclusive measure for imaging and the most comprehensive measure for pathology, respectively. ROC AUCs for composite-cerebellum/and contralateral-pons/Bielschowsky silver were nearly as large.

Based on the sum of sensitivity and specificity, BIE was a better tool for predicting the Bielschowsky pathology findings than was quantitation with SUVR (in 6 of the 6 SUVR/SoT pairs) but very similar to both quantitation with SUVR and BIE using the overall pathology judgment (i.e., high sensitivity and high specificity); the converse was true for thioflavin S (less sensitive and similarly specific) and 4G8 (substantially less sensitive and usually more specific). Overall, based on the sum of sensitivity and specificity, BIE and quantitation with SUVR appeared to be tied.

No particular advantage to using one SUVR reference region over the other (i.e., cerebellum or pons) was apparent. However, for all SUVR types, SUVR cut-off criteria using pons as the reference region were unexpectedly found to be less than 1. From the observed optimal cut-off values of approximately 0.4 (pons as reference region) and approximately 1.2 (cerebellum as reference region) in this set of iNPH patients, one would predict that on average, much more Aβ was deposited in the pons than in the cerebellum. Whereas in AD, enough Aβ for detection with tracer does not appear in the cerebellum or pons until Thal Phase 5 [37]. The significance of this finding will need to be clarified in future research.

We should note that a post-hoc Spearman correlation analysis was performed between cognitive status (Mini-Mental State Examination [MMSE] scores, for which individual subject values were previously published [32]), and measures for all of the pathologic markers of amyloid, including Bielschowsky, 4G8, thioflavin S, and overall pathology judgment. No statistically significant correlation was found, consistent with previous results that found no significant correlation between an imaging marker for amyloid load ([^11^C]PIB binding potential with PET) and MMSE [46].

While neocortical pathological Aβ changes in affected iNPH patients are similar to those in AD, and it is tempting to apply findings from one condition to the other, it is important to recognize that our understanding of the molecular biology of amyloid is incomplete [47]. We biopsied only cortical samples for use as our SoT. While a reduction in brain tissue is characteristic of both AD and iNPH, the reduction is due to atrophy in AD, whereas the sulci are less enlarged relative to the degree of ventricular dilatation in iNPH. However, the fact that the overall pathology SoT fared so well in the results may indicate that neocortical areas where amyloid was present in our iNPH patients might have been mimicking where Aβ pathological changes are seen in AD, i.e., widespread vs. focal. Others confirm the validity of diagnosis from a single cerebral biopsy sample [48].

To put the 4G8 antibody data into context, variable processing of the membrane-bound amyloid precursor protein (APP) produces Aβ and other APP fragments including p3 [47]. Aβ is a 39-43 amino-acid peptide; the 4G8 antibody detects amino acids 17-23 [49] or 17-24 [37]. The morphology of deposits in AD brain detected with 4G8 have been described with photomicrographs in exquisite detail as fleecy (fine fibrillar [49]), lake-like, and subpial band-like amyloid; diffuse and cored plaques; and core-only (burnt-out [50]) plaques, as well as white matter plaques; some of which types are further sub-divided into neuritic and non-neuritic [51]. From neocortex (Thal Phase 1), Aβ as detected using silver stain and 4G8 appears progressively over time in specific connected brain structures in an ordered anterograde sequence [51,37].

Ikonomovic et al. demonstrated co-localization in an AD brain of 6-CN-PiB (thioflavin T derivative) and thioflavin S to cored plaques and core-only plaques in temporal cortex. Thioflavin S was not as selective and also stained numerous neurofibrillary tangles in this region, whereas 6-CN-PiB detected only an occasional isolated tangle [22].

Silver staining and IHC for Aβ provide complementary information. Silver, which stains both plaques and tangles (which appear in neuritic plaques), renders stable and reproducible results [52] and is recommended for quantitation of neuritic plaques [38,43]. However, ‘argyrophilia’ is not a homogeneous phenomenon with respect to amyloid [51], and other subtle lesion-dependent variations between silver methods have been described [52].

While the newest NIA-AA criteria for the neuropathologic assessment of AD [38] also recommend IHC for ‘Aβ score’ (not plaque count) and refer to Thal [37], the NIA-AA criteria stop short of specific Aβ IHC reagent recommendations. Technical uncertainties with respect to Aβ IHC standardization may preclude its use as a standard for neuropathologic diagnosis, at least when deposits are *quantified* as with histological diagnostic criteria for AD [52]. For the neuropathologic changes of AD, the new criteria recognize the *qualitative* importance of the location and morphology of Aβ deposits as separate and different from quantification by neuritic plaque counts as in the CERAD scheme [38] or, by extension, as in the percentage area positivity we measured with 4G8 in this study of iNPH cortical biopsies.

Spillantini et al. reported finding “a substantial increase in the number of stained structures and the intensity of staining” with 4G8 after pre-treatment with formic acid [53], although data relative to no pre-treatment were not shown. Shankar et al. believe that formic acid solubilizes insoluble Aβ plaque cores isolated from human AD brains and releases their constituent dimers and monomers [54].

Plaques, composed largely of fibrillar Aβ, are dynamic structures and likely act as local reservoirs of smaller diffusable Aβ oligomers thought to be in equilibrium with plaques [55]. Disruption of the electrophysiological and microanatomical correlates of memory formation (i.e., inhibition of long-term potentiation, facilitation of long-term depression, reduced dendritic spine density, synapse loss) are associated with the smaller, soluble Aβ species [55], which are suspected of eventually [56] tipping the gain-of-toxic-function [47] past steady-state in favor of Aβ conglomeration and towards subsequent downstream events. Importantly, this paradigm provides that plaques may be present in cognitively (i.e., phenotypically) normal individuals. Clinically identifying patients on this cusp (cognitively normal/abnormal) and monitoring their disease progression (i.e., locating amyloid plaques in living patients) is of tremendous relevance and urgent importance to the testing of drugs that exploit the molecular biology of Aβ for the treatment of AD.

In this study, we found that while the numbers of FN and FP results were low (lowest for the overall pathology SoT), for each of the 3 stains separately, the proportions of patients with FP was substantially greater than the proportions of patients with FN. Interestingly, in our experience based on a large autopsy study of [^18^F]flutemetamol (68 brains, 43 Aβ positive, 25 Aβ negative) [report in process], cases with sparse to moderate diffuse and neuritic cortical plaques (Bielschowsky silver stain) may lead to FN or FP PET readings (similar to those discussed in a recent review [57]). This ‘cusp’ phenomenon may reflect the (currently unsettled) rest of the story of the molecular biology of Aβ, what triggers and perpetuates its neuronal anterograde trek [51,37] and under what conditions (including the inflammatory component of the pathology outside the scope of this report), and what determines the point at which the recognizable phenotype becomes apparent (i.e., the presumed point of irreversible functional damage).

The 4G8 antibody has been used before in association with amyloid PET ligand uptake [33,27]. The 4G8 component of our analysis can be interpreted both in an isolated manner and in context. We questioned the acceptability of pooling the 4G8 data from Study A (obtained using an antibody dilution of 1:500) with that from Studies B, C, and D (obtained using an antibody dilution of 1:100). We noticed that Clark et al. used 4G8 at a dilution of 1:2000 and we think reported similar correlations (the use of Bonferroni ρ was unclear) to what we previously reported for our pooled data (using Pearson’s r) [32] in their [^18^F]florbetapir PET autopsy study in subjects with and without AD or other age-related pathologies [58,33]. And, Thal [49,37] and Braak [59] used a dilution of 1:5000. In a systematic inter-laboratory study, Alafuzoff et al. recommended that in order to achieve reproducible results with 4G8 IHC, a dichotomized assessment (they suggested present/absent) rather than quantification should be applied [60]. Our raw data showed that in Study A 4/7 biopsy samples had 4G8 present; in Studies B, C, and D, respectively, 6/9, 13/15, and 16/16 biopsy samples had 4G8 present. Given the small sample sizes, it is impossible to know how different ‘4G8 present/absent’ in our studies truly was, and therefore whether or not pooling of data on this basis is indicated.

Our data showed that the 4G8 SoT did not perform as well as the other SoTs in terms of the sum of sensitivity and specificity; it showed good sensitivity and poor specificity (indicating too many FP). Tissue preparation may have been the reason for this, in that the threshold for abnormal may not have been optimized. If the threshold for abnormal were set lower than the 2.5% we used, sensitivity would have decreased and specificity would have increased. The fact that the 4G8 dichotomous assessment did not match the overall pathology SoT in many cases (i.e., 4G8 resulted in the most overall pathology misclassifications) supports the interpretation that the selected threshold was not optimal.

In a separate analysis (Study C) of iNPH patients included in this pooled analysis and who also underwent [^11^C]PiB PET imaging, ipsilateral, contralateral, and composite SUVRs for both [^18^F]flutemetamol and [^11^C]PiB correlated significantly with Aβ biopsy specimen levels evaluated by 4G8, thioflavin S, and Bielschowsky silver stain [30]. Our findings using the pooled [^18^F]flutemetamol data in iNPH patients are also consistent with findings for [^18^F]florbetapir where a correlation was shown between PET brain labeling and grey matter plaque density not only by 4G8 as alluded to above, but also as measured by silver stain at autopsy in subjects with and without AD or other age-related pathologies [58,33]. To our knowledge, no biopsy or autopsy data for [^18^F]florbetaben have been published yet.

Whereas our study was limited by the logistical constraints associated with the collection of comparatively primitive neuropathologic data from clinical trials at multiple sites in the setting of rapidly changing (increasingly refined) pathologic diagnostic criteria, we propose that an ideal experiment might improve upon a similar basic study design to that of Ikonomovic et al. [22] and include the following elements: multiple patients with AD and brain [^18^F]flutemetamol imaging near life’s end, followed by post mortem examination of adjacent sections treated with cyano-flutemetamol vs. e.g., IHC for selected Aβ epitopes and tau epitopes (plus routine conventional staining), with the Aβ and neurofibrillary tangle pathology results described according to current nomenclature for neuropathologic changes of AD [38]. Other desirable experimental elements include a selected battery of sections of brains associated with all phases of AD severity, sections thick enough to allow confocal microscopy through entire cells, collection of information with respect to individual genetic risk association factors, thorough history and timeline of medical conditions with any inflammatory component, duration of cognitive impairment, and results of recent neuropsychiatric assessments. The importance of careful archiving of tissue samples and associated patient data to test for future findings cannot be overstated. Paradoxically, owing to the relative rarity of appropriate post mortem handling and disposition for these purposes, the human brain may be as valuable an asset in death as it is in life.

The performance characteristics and diagnostic efficacy of the Aβ ligands when used alone, and more recently when used with other imaging modalities (e.g., structural MRI and fluorodeoxyglucose PET) [e.g., [42,61] or in conjunction with the assessment of the well known AD risk variant of apolipoprotein E (ϵ4 allele) [e.g., [62], have been described in over a decade of literature. ApoE type has not yet added clinically useful diagnostic information in conjunction with imaging; however, algorithms which include multiple biomarkers have been clearly shown to increase the power of studies and reduce the number of patients required to demonstrate the statistical significance of findings [61] and are encouraged by the Food and Drug Administration [63] and the Alzheimer’s Disease Neuroimaging Initiative (http://www.nia.nih.gov/research/dn/alzheimers-disease-neuroimaging-initiative-adni), which freely shares data. While we did not pre-specify the collection any CSF biomarker data in our 4 clinical trials (and ApoE genotype was published for only 18 subjects in our pooled dataset [31]), the relationship of certain CSF biomarkers to brain biopsy findings was recently described for a large series of patients (53 iNPH, 26 AD, and 23 other) at Kuopio University in Finland; quantified biopsy Aβ load showed a negative correlation with both ventricular and lumbar CSF Aβ_42_ while levels of Aβ_38_ and Aβ_40_ showed no correlation [64]. In the near future we hope to see clinical imaging with Aβ ligands combined with assessments of other recently identified AD risk genes (up to and including IHC for their protein products at autopsy) such as for specific variants of clusterin (CLU, Apo J), complement component receptor 1 (CR1), phosphatidylinositol binding clathrin assembly protein (PICALM) [61], until a more powerful algorithm is achieved that will almost certainly inform the identity of one or more useful drug targets (drugs) for the slowing, prevention, and/or arrest of this ultimately devastating pathology.

In summary, both quantitative assessment and BIE of [^18^F]flutemetamol images in this series of iNPH patients showed good agreement with cortical biopsy histopathology. The primary diagnostic effectiveness (as measured by ROC AUC, Youden index, and sum of sensitivity and specificity) for [^18^F]flutemetamol PET was best when the composite SUVR measure using cerebellum as the reference region was paired with the overall pathology SoT.

## Competing interests

PS, AS, KH, CJB, and IDG are employees of GE Healthcare. DFW has received contract funds via JHU only from GE Healthcare.

## Authors’ contributions

VL, JOR, DFW, DAW, JQT, PFS, AS, KH, and IDG contributed to the conception, organization, and execution of the research project as well as review and critique of the manuscript. MS takes responsibility for the accuracy of the data analysis as well as review and critique of the manuscript. IDG also had responsibility for the integrity of the data and the final decision to submit the manuscript for publication, led the research team, obtained approval from the sponsor, and submitted the manuscript for publication. All authors read and approved the final manuscript.
